# Zinc lysine and selenium yeast can effectively improve the reproductive performance of Northeast white geese

**DOI:** 10.1016/j.psj.2025.104867

**Published:** 2025-01-31

**Authors:** Jingchun Li, Heze Feng, Yulun Song, Hechuan Wang, Yingying Dong, Han Li, Qing Guo, Shengjun Liu, Yanbing Li

**Affiliations:** a College of Animal Science & veterinary medicine, Heilongjiang Bayi Agricultural University, Daqing, Heilongjiang 163319, PR China; b Key Laboratory of Exploration and Innovative Utilization of White Goose Germplasm Resources in the Cold Region of Heilongjiang Province, #2 Xinyang Road, New Development District, Daqing, Heilongjiang, PR China 163319

**Keywords:** Goose, Selenium yeast, Zinc lysine, Performance of reproduction

## Abstract

The aim of this study was to investigate the effects of dietary supplementation with selenium yeast (YS) and zinc lysine (ZL) on goose semen quality, testicular tissue structure, reproductive hormone levels, antioxidant capacity and egg fertilization rate. A total of 24 male and 72 female geese, all 180-days-old, were selected and divided into four treatment groups, each with a consistent body weight. Each treatment group had six male and 18 female geese (1:3 ratio of male to female). The control (Ⅰ) group was fed a basal diet. The YS (Ⅱ) group was fed a basal diet supplemented with 0.3 mg/kg of selenium-yeast. The ZL (Ⅲ) group was fed a basal diet supplemented with 106.05 mg/kg of zinc-lysine. The YS + ZL (Ⅳ) group was fed a basal diet supplemented with 0.3 mg/kg of selenium-yeast and 106.05 mg/kg of zinc-lysine. The experimental period lasted for 91 days. The results showed that supplementation of 0.3 mg/kg YS and 106.05 mg/kg ZL significantly increased sperm motility (*P* < 0.05), and significantly increased VAP, VSL, VCL, ALH, LIN, MAD and STR (*P* < 0.01) compared with the control group. Compared with the control group, dietary supplementation of YS and ZL increased the number of germ cells and Johnson score in groups Ⅱ, Ⅲ and Ⅳ (*P* < 0.01), and the number of spermatogonia and sertoli cells were significantly different from the control group (*P* < 0.05). Dietary supplementation of YS and ZL could increase the reproductive hormone level (*P* < 0.05) and serum antioxidant capacity (*P* < 0.05), and the fertilization rate showed a certain promoting trend (*P* = 0.09). In conclusion, dietary supplementation of 0.3 mg/kg YS and 106.05 mg/kg ZL can improve semen quality, promote testicular development, maintain stability of the fertilization rate, and increase reproductive hormone levels and the serum antioxidant capacity of geese.

## Introduction

Selenium and zinc are essential trace elements that play important roles in animal growth, immunity, anti-oxidation and reproduction pathways (Bedwal and Bahuguna, 1994; Ferencík and Ebringer, 2003; Steinbrenner and Klotz, 2020). Normal body metabolism will produce free radicals. However, the loss of balance between antioxidant and oxidative substances in the antioxidant system can cause the accumulation of free radicals in body tissues, resulting in lipid peroxidation and cell membrane damage (Yin et al., 2011). Selenium participates in metabolic processes by forming the active center of enzymes, reducing oxidation and eliminating free radicals, and can be used to prevent and treat various diseases caused by nutritional deficiency diseases and free radical accumulation (Wang et al., 2017; Bjørklund et al., 2022; Genchi et al., 2023). However, the bioavailability of inorganic selenium is low, its dosage cannot be effectively controlled, and excessive amounts in the diet is toxic to animals (Mahima et al., 2012). Selenium yeast (YS), a biological form of selenium, can eliminate the toxic side effects of chemical selenium (such as sodium selenite) on organisms, and allows selenium to be absorbed and fully utilized by organisms more efficiently (Rayman, 2004). In the current study of inorganic selenium forms, sodium selenite is still one of the main choices, while selenium yeast is still the main organic form (Ferrari et al., 2023). Selenium yeast can effectively alleviate oxidative damage by regulating different signaling pathways, increasing the activity of antioxidant enzymes, reducing the content of inflammatory factors, regulating the expression of apoptosis-related proteins, and reducing the accumulation of mycotoxins and heavy metals in the body (Sun et al., 2023). Studies have shown that YS plays a protective role in the testis against aluminum-induced toxicity by maintaining ion homeostasis via the reduction of oxidative stress and nitric oxide production (Cao et al., 2020). Dietary supplementation of YS can effectively improve sperm motility, plasma membrane function and integrity, and ejaculate volume. It can also effectively improve fertility and hatching rate, as well as the thickness of seminiferous epithelium and the diameter of seminiferous tubules (Sabzian-Melei et al., 2022). Zinc, an essential trace element, is a cofactor for many metalloenzymes required for cell membrane repair, cell proliferation, growth, and immune system function, and is essential for growth, development, and maintenance of immune function. Zinc deficiency leads to the development of skin lesions, growth retardation, impaired immune function, and impaired healing. Its influence spans all organs and cell types, accounts for approximately 10 % of the human proteome, and contains hundreds of key enzymes and transcription factors (Lin et al., 2017; Read et al., 2019). Zinc is involved in the metabolism of proteins, amino acids, nucleic acids, fats and carbohydrates in animals, and is also related to the activity of insulin, glucagon, prostaglandins and gonadotropins. Zinc is necessary for physiological functions, such as reproduction, immunity, coagulation and biofilm stability. However, long-term high-dose zinc supplementation can lead to copper deficiency or anemia (Saper and Rash, 2009; Muhamed and Vadstrup, 2014; Baltaci et al., 2018). In reproduction, the functions and cellular mechanisms of zinc primarily involve sperm motility, capacitation and acrosome exocytosis, all of which are crucial for successful fertilization (Allouche-Fitoussi and Breitbart, 2020). Zinc lysine (ZL) is a trace element amino acid chelate, which is used as a feed and nutritional additive. It has the advantages of moderate chemical stability, high biological potency, special absorption mode and metabolic pathway, and has dual nutritional and therapeutic effects. Studies have shown that ZL has great potential to reduce the toxicity of heavy metals without damaging the normal growth and development of plants (Zaheer et al., 2019), suggesting it may also be applicable to animal growth and reproduction experiments. Lysine is the first limiting amino acid, and its trace element chelates have special uses and broad market prospects (Fouad, 1976). ZL has the advantages of a five-membered ring chelating structure with neutral intramolecular charge, stable chemical properties, good palatability and high bioavailability, and provides an ideal nutritional fortifying agent and feed additive (Ward et al., 1997; Datta et al., 2001). The lysine and glutamate zinc chelate is a bioavailable zinc source comparable to the standard inorganic zinc source, and this additive is effective in meeting the zinc requirements of poultry, as well as many other animal species (EFSA Panel on Additives and Products or Substances used in Animal Feed (FEEDAP) et al., 2019).

At present, there are many reports on the performance and meat quality of geese through different feeding methods (Yan et al., 2023), but there are relatively few physiological and biochemical studies, and there are few reports on the effects of selenium and zinc on gander reproduction. Therefore, the current study aimed to investigate the effects of selenium and zinc on the reproductive performance of the gander. The specific goals of this study were to explore the effects of YS and ZL on goose semen quality, testicular tissue structure, reproductive hormone levels, antioxidant capacity and fertilization rate of eggs, to provide a theoretical basis for understanding the role of trace elements zinc and selenium on the growth and breeding of geese.

## Material and methods

### Ethics approval

This study was approved by the Laboratory Animal Ethics Committee of the Heilongjiang Bayi Agricultural University. (DWKJXY2023073)

### Ganders and experimental design

The 180-day-old ganders was selected as the test goose, which was provided by Anda Lion Seed Goose Breeding Co., Ltd. (Daqing, China). The feeding management follows the provisions of "meat goose standardized breeding operation manual". Using the orthogonal trial design method, twenty-four 180-day-old male geese and seventy-two female geese were selected and divided into 4 treatment groups according to the principle of consistent body weight. The basal diet (group I), YS content of 0.3 mg/kg (group Ⅱ), ZL 106.05 mg/kg (group Ⅲ), and YS 0.3 mg/kg + ZL 106.05 mg/kg (group Ⅳ) test period was 91 days. Each treatment group consisted of 6 male geese and 18 female geese, maintaining a male-to-female ratio of 1:3. For male geese, three replicates were set, with two male geese in each replicate; for female geese, six replicates were arranged, with three female geese in each replicate. During the test period, ventilation was frequent, an artificial light replacement system was adopted, which provided light throughout the day, animals had free access to diet and drinking water, and the rest was carried out according to routine feeding management. YS was provided by Zhejiang Shenyou Biotechnology Co., Ltd. with a purity of ≥97 % and 98.9 % organic selenium. ZL was provided by Ningbo Yingqian Technology Co., Ltd., purity ≥95 %, containing 15 % elemental zinc. The addition amounts of YS and ZL referred to previous relevant research papers (Wang et al., 2009; Chen et al., 2013). Therefore, the selenium content in control group (Ⅰ), YS group (Ⅱ), ZL group (Ⅲ) and YS + ZL group (Ⅳ) were 0.3 mg/kg, 0.6 mg/kg, 0.3 mg/kg and 0.6 mg/kg, respectively. The zinc content was 90 mg/kg, 90 mg/kg, 105.91 mg/kg and 105.91 mg/kg, respectively. The preparation of the experimental diet follows the NRC nutritional standards (“Nutrient Requirements of Poultry: Ninth Revised Edition, 1994 | The National Academies Press,”). The composition and nutrients of the basal diet are shown in Table 1.

**Table 1:** Composition and nutrient levels of basal diets (air-dry basis) %.

Ingredients	Content	Nutrient levels	Content
Corn	61.50	ME/(MJ/Kg)^2^	10.92
Soybean meal	25.00	Crude protein	16.02
Rice bran	7.00	Crude fibre	5.60
Wheat bran	3.00	Calcium	0.72
NaCl	0.30	Phosphorus	0.54
Premix^1^	1.00	Lysine	0.80
DL-Met	0.10	Methionine	0.33
CaHPO_4_	1.10		
Mountain flour	1.00		

1 Premix was provided per kg of diet: Zn 90 mg, Cu 6 mg, Mn 85 mg, Fe 80 mg, I 0.42 mg, Se 0.3 mg, vitamin A 8.000 IU, vitamin E 100 mg, vitamin D 1600 IU.

2 ME was a calculated value, while other values were quantified through measurement.

### Sample collection and processing

At the 1st, 3rd, 5th, 7th and 11th week of the experiment, three male geese in each group were selected for artificial sperm collection via massage. From the 1st week to the 13th week of the experiment, eggs were collected every day, disinfected and incubated in an incubator (BY-1232, Benying Environmental Protection Technology Co., Ltd., Shandong, China) at 37.5℃. On the 90th day of the experiment, three experimental ganders with similar weight were selected from each treatment group, a total of 12 experimental ganders, 5 mL blood samples were collected from the lower vein of each goose wing, and placed in a collecting vessel. Serum was prepared by centrifugation at 1000 × g for 15 min and stored at −20°C. On the 91st day of the experiment, three geese were randomly selected from each group and euthanized, and a total of 12 freshly collected left testis tissues were fixed in 4 % paraformaldehyde for more than 24 h prior to tissue processing and sectioning.

### Measurement of semen quality

Immediately after semen collection, samples were placed in a metal bath at 39°C. Ten μL of semen was transferred to 39°C heat preservation culture medium by a pipetting gun, and then put on a glass slide. The Computer-Aided Sperm Analysis system in an automatic sperm analyzer (MD06200C, Songjing Tianlun Biotechnology Co., Ltd., Nanjing, China) was used to analyze goose sperm viability, motility, average path velocity (VAP), straight line velocity (VSL), curvilinear velocity (VCL), amplitude of lateral head displacement (ALH), linearity (LIN), moving angle (MAD), sperm track straightness (STR), beat cross frequency (BCF) and wobble (WOB) in multiple fields. The proportion of sperm with different motility levels in the total number of sperm was calculated, and semen quality was comprehensively evaluated based on other motility parameters.

### Measurement of morphological parameters of testicular tissue structure

On the 91st day, three male geese were randomly selected and euthanized from each group, and a total of 12 geese were used for left testis collection and tissue preparation. Freshly collected left testis tissue was fixed in 4 % paraformaldehyde for more than 24 h, then the tissue was removed and trimmed in a fume cabinet with a knife, and placed in a dehydration box. Different concentrations of alcohol, alcohol benzene, xylene and wax were used for gradient dehydration and wax immersion, and the tissue was finally placed on a microtome for sectioning at a thickness of approximately 4–6 μm, with at least three slices from each testis. The prepared paraffin sections were dried, deparaffinized, and stained with hematoxylin and eosin. The stained sections of goose testis were then placed under a microscope (BX53, OLYMPUS, USA) for observation and image acquisition. Image Pro plus 6.0 software was used to measure the number of spermatogenic cells in the testis. The maturation of spermatogenic cells in the seminiferous epithelium was classified according to Johnsen's score.

### Measurement of reproductive hormone level and serum antioxidant capacity

Serum reproductive hormone level and antioxidant indexes were measured on day 90, reproductive hormone including luteinizing hormone (LH), follicle stimulating hormone (FSH) and testosterone (T), and the kit is provided by Enzymelinked Biotechnology Co., Ltd. (Shanghai, China). Antioxidant indexes include superoxide dismutase (SOD), glutathione peroxidase (GSH-Px), total antioxidant capacity (T-AOC), catalase (CAT), malondialdehyde (MDA), and the kit is provided by Suzhou Grace Biotechnology Co., Ltd. (Suzhou, China).

### Measurement of fertility rate of goose eggs

The hatching temperature of goose eggs is 37.5℃ and the humidity is 65 %. The eggs are turned every 6 h from 0 to 15 days, while egg turning is stopped from 15 to 30 days. Beginning on the 15th day, the hatching temperature is adjusted to 36.5℃ and the humidity is increased to 70 %. The hatching eggs were disinfected by spraying with mesitylene trimethyl ammonium chloride solution (Huatian Pharmaceutical Co., Ltd., Inner Mongolia, China). From 0 to 7 days, unfertilized eggs were screened out, and the fertilization rate of each treatment group was calculated at 30 days (fertilization rate = number of fertilized eggs/number of hatched eggs ×100 %).

### Statistical analysis

SPSS 26.0 statistical software was used for statistical analysis of the research data. Data results are expressed as mean ± standard error. SPSS software was used for homogeneity test of variance analysis. LSD, Waller-Duncan and Tukey s-b (K) tests of one-way ANOVA were used for data between different treatment groups. *P* < 0.05 was considered as significant difference level, and *P* < 0.01 was considered as extremely significant difference level. Fig. were produced by data visualization software GraphPad Prism 10.

## Results

### Semen quality

From Table 2, it can be seen that the sperm viability of group Ⅰ is significantly different from that of group Ⅱ, group Ⅲ and group Ⅳ (*P* < 0.05). There was no significant difference in sperm motility, BCF and WOB between each treatment group. The VAP, VCL, ALH in group Ⅱ and Ⅳ were significantly different from those in group Ⅰ and Ⅲ (*P* < 0.01). Compared with group I, there were significant differences in VSL in groups II, III and IV (*P* < 0.01). Compared with the other three groups, the differences of LIN and STR in group Ⅲ were extremely significant (*P* < 0.01). Compared with group I, III and IV, the MAD in group II showed significant difference (*P* < 0.01), and the difference was more obvious with group III.

**Table 2:** Effects of selenium yeast and zinc lysine supplementation on semen quality of gander.

Item	Ⅰ	Ⅱ	Ⅲ	Ⅳ	*P*-value
Sperm viability(%)	43.63±1.33^b^	58.56±2.72^a^	60.04±4.82^a^	58.57±4.71^a^	<0.05
Sperm motility(%)	36.01±1.06	45.01±2.24	45.03±3.62	43.93±3.54	0.11
VAP(μm/s)	69.26±4.10^B^	140.48±6.58^A^	56.33±4.52^B^	151.56±12.97^A^	<0.01
VSL(μm/s)	133.04±4.64^B^	187.21±8.74^A^	195.78±15.72^A^	200.10±16.95^A^	<0.01
VCL(μm/s)	98.66±5.84^B^	200.09±9.37^A^	80.25±6.44^B^	215.88±18.48^A^	<0.01
ALH(μm)	29.39±1.74^B^	59.61±2.79^A^	23.91±1.92^B^	64.32±5.51^A^	<0.01
LIN	1.36±0.05^B^	0.94±0.00^C^	2.44±0.00^A^	0.92±0.00^C^	<0.01
MAD(°/s)	1.64±0.05^B^	2.11±0.04^A^	0.35±0.00^C^	1.32±0.22^B^	<0.01
STR	1.94±0.07^B^	1.34±0.00^C^	3.48±0.00^A^	1.32±0.00^C^	<0.01
BCF(Hz)	17.87±0.39	16.58±0.33	17.77±0.42	17.32±0.47	0.13
WOB	0.70±0.00	0.70±0.00	0.70±0.00	0.70±0.00	1.00

Abbreviations: VAP, average path velocity; VSL, straight line velocity; VCL, curvilinear velocity; ALH, amplitude of lateral head displacement; LIN, linearity; MAD, moving angle; STR, sperm track straightness; BCF, beat cross frequency; WOB, wobble.

Peer data with different capital letters on shoulder labels indicated that the difference was extremely significant (*P* < 0.01), and without or with the same capital letters indicated that the difference was not significant (*P* > 0.01). Peer data with different lowercase letters on shoulder labels indicated that the difference was significant (*P* < 0.05), and no labeling or containing the same lowercase letters indicated that the difference was not significant (*P* > 0.05).

### Morphological parameters of testicular tissue structure

**Fig. 1:**
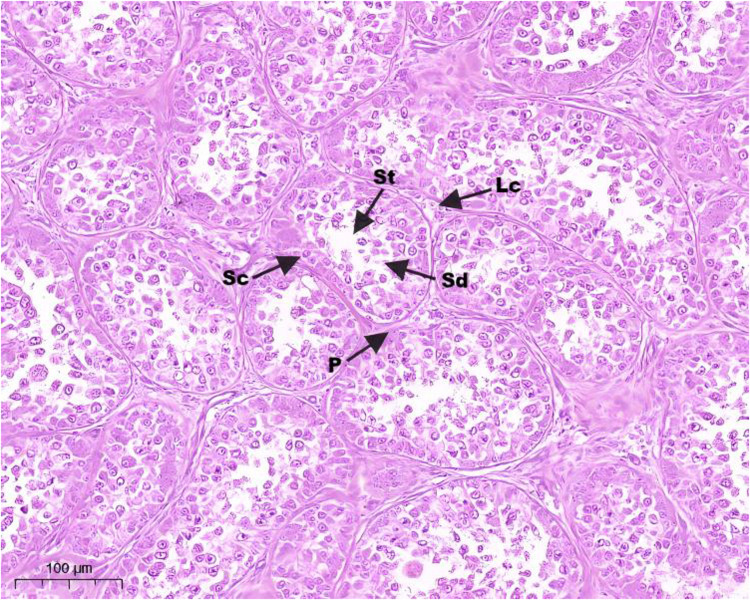
Results of H&E staining of testicular tissue from gander in the control group. Note: St: Seminiferous tubule, Sc: Sertoli cells, Sd: spermatid, Lc: Leydig cells, P: Spermatogonia S: Sperm. The figure below is the same.

**Fig. 2:**
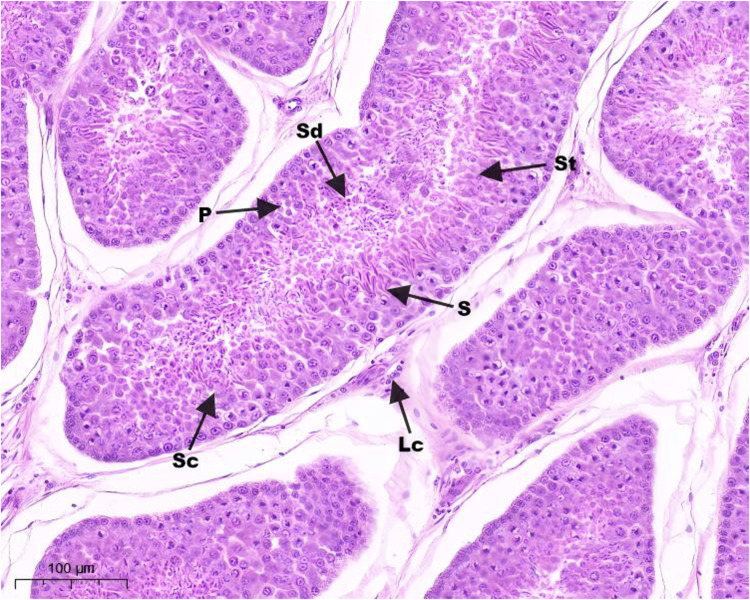
Results of H&E staining of testicular tissue from gander in the selenium yeast group.

**Fig. 3:**
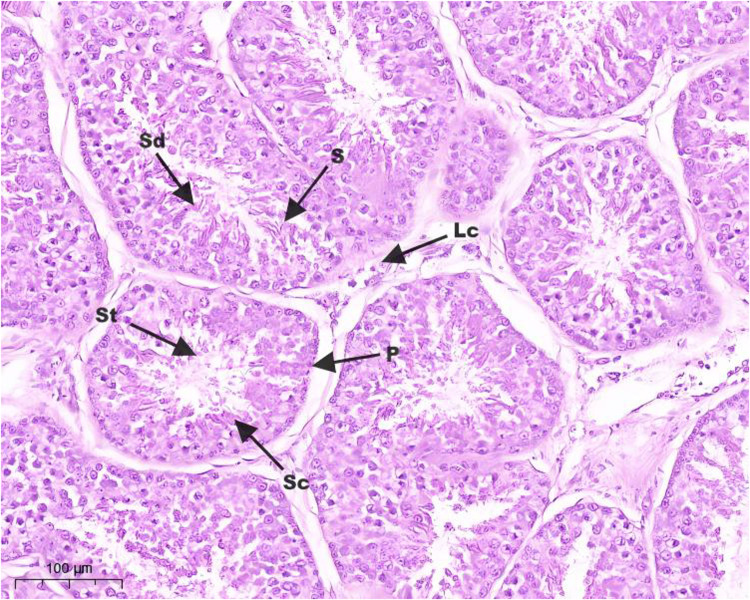
Results of H&E staining of testicular tissue from gander in the zinc lysine group.

**Fig. 4:**
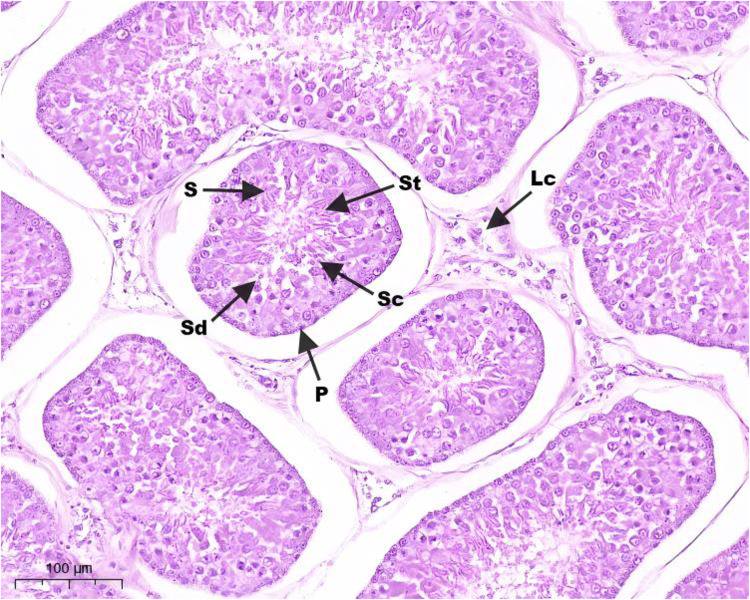
Results of H&E staining of testicular tissue from gander in the selenium yeast + zinc lysine group.

Hematoxylin and eosin (H&E) staining results of testis tissue of Northeast White Goose are shown in the figure. In the control group, leydig connective tissue is less, and 3–5 layers of spermatogenic cells and sertoli cells are arranged in spermatogenic tubules (Fig. 1). In YS group (Fig. 2), ZL group (Fig. 3) and YS + ZL group (Fig. 4), there are many connective tissues in testis. The seminiferous tubules are composed of 4-6 layers of spermatogenic cells and columnar sertoli cells. The sertoli cells and spermatogonia are distributed around the basement membrane of seminiferous tubules, and sperm are distributed in clusters in seminiferous tubules. As shown in Fig.s A, B, Fig. C, Fig. D, compared with the control group, the number of germ cells and Johnson score in YS group, ZL group and YS + ZL group were significantly different (*P* < 0.01), and the number of spermatogonia and sertoli cells in YS group, ZL group and YS + ZL group were significantly different from those in the control group (*P* < 0.05).

**Fig. A:**
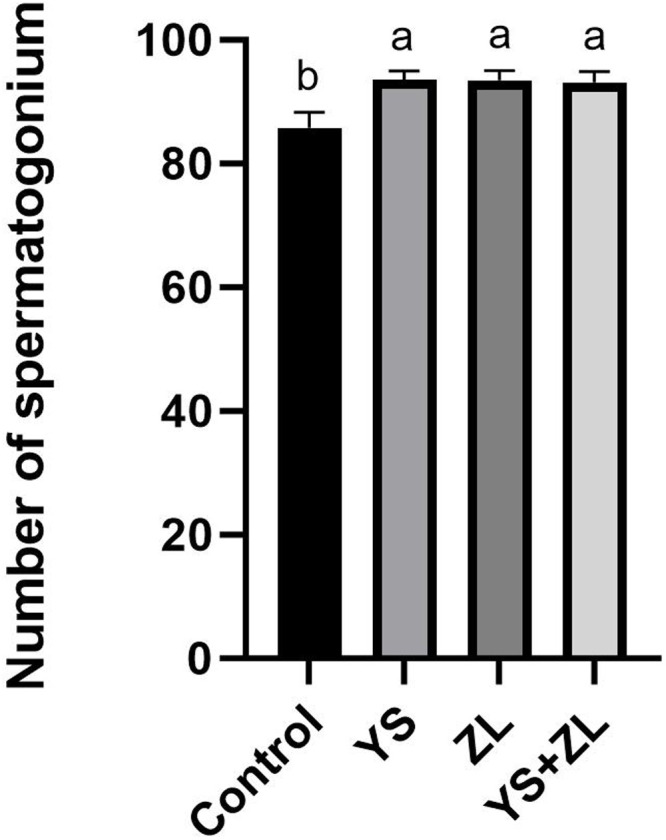
Effects of supplemental selenium yeast and zinc lysine in diets on the number of spermatogonium cells in the testis of gander. Note: Data with different capital letters indicated a significant difference (*P* < 0.01), while data with no marked or the same capital letters indicated no significant difference (*P* > 0.01). Data with different lowercase letters indicated a significant difference (*P* < 0.05), while data with no marked or the same lowercase letters indicated no significant difference (*P* > 0.05). The figure below is the same.

**Fig. B:**
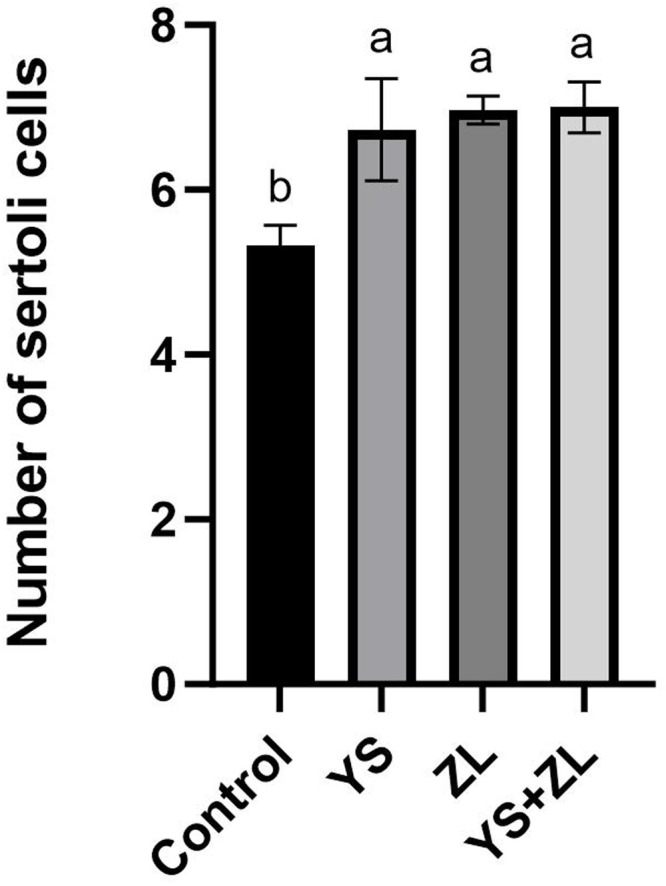
Effects of supplemental selenium yeast and zinc lysine in diets on the number of sertoli cells in the testis of gander.

**Fig. C:**
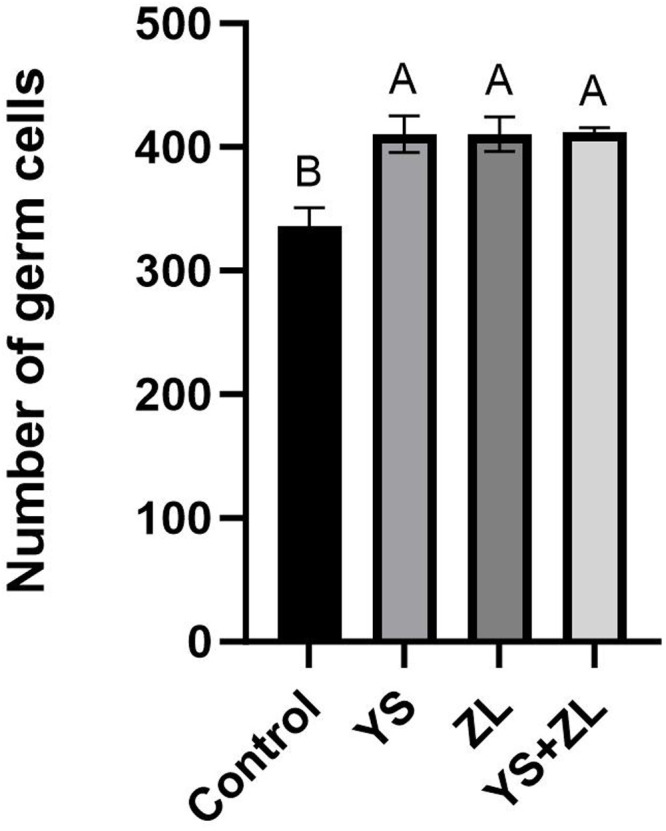
Effects of supplemental selenium yeast and zinc lysine in diets on the number of germ cells in the testis of gander.

**Fig. D:**
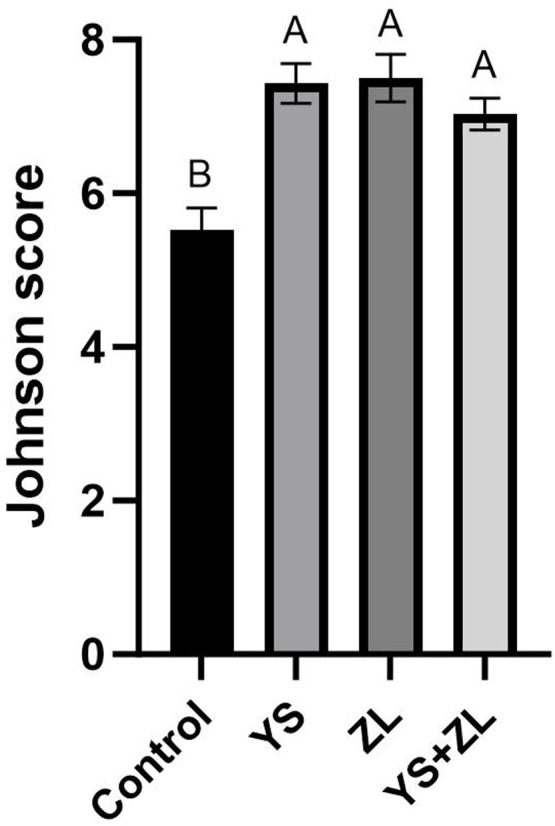
Effects of supplemental selenium yeast and zinc lysine in diets on Johnsen's score in the testis of gander.

### Reproductive hormone level and serum antioxidant capacity

As can be seen from Fig. E, Fig. F, Fig. G, the levels of LH, T and FSH in groups II, III and IV were significantly higher than those in the control group (*P* < 0.05). It can be seen from Table 3 that the MDA content in the serum of male geese in group Ⅰ was significantly higher than that in groups II, III and IV (*P* < 0.01). Compared with the control group, the concentrations of GSH-Px and SOD in groups Ⅱ, Ⅲ and Ⅳ increased significantly (*P* < 0.05). The concentrations of T-AOC and CAT in groups Ⅱ, Ⅲ and Ⅳ were significantly different from those in the control group (*P* < 0.01).

**Fig. E:**
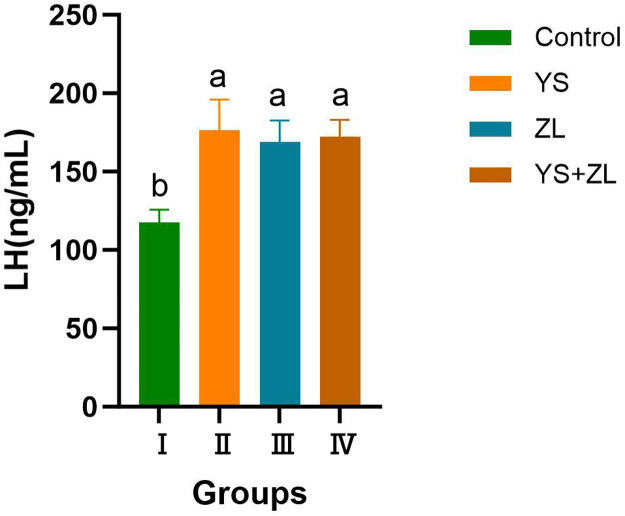
Effects of selenium yeast and zinc lysine supplementation on LH of gander.

**Fig. F:**
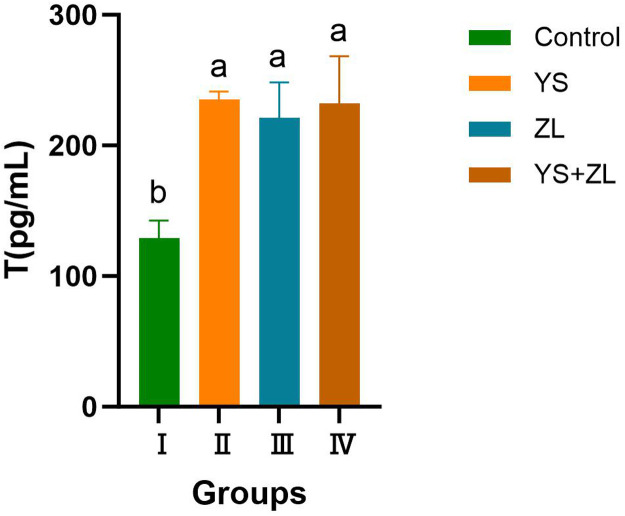
Effects of selenium yeast and zinc lysine supplementation on T of gander.

**Fig. G:**
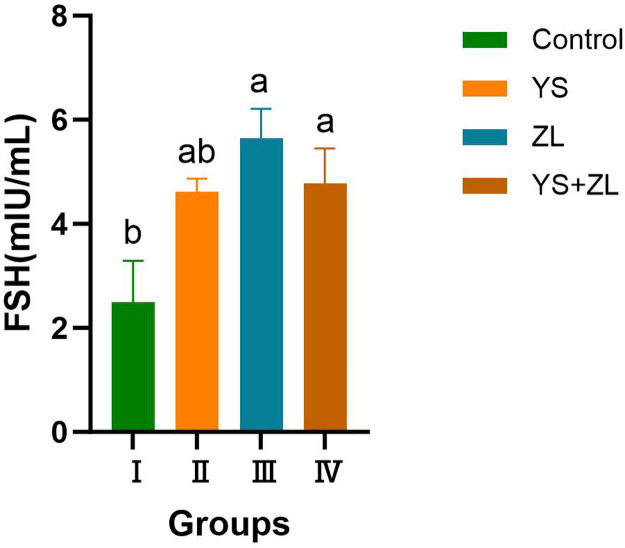
Effects of selenium yeast and zinc lysine supplementation on FSH of gander.

**Table 3:** Effects of selenium yeast and zinc lysine supplementation on serum antioxidant indexes of gander.

Item	Ⅰ	Ⅱ	Ⅲ	Ⅳ	*P*-value
GSH-Px(U/mL)	399.49±17.38^b^	469.80±19.56^a^	460.00±12.27^a^	496.57±13.98^a^	<0.05
MDA(nmol/mL)	0.76±0.07^A^	0.47±0.05^B^	0.37±0.04^B^	0.39±0.02^B^	<0.01
T-AOC(μmol/mL)	2.04±0.03^B^	2.45±0.01^A^	2.44±0.05^A^	2.46±0.06^A^	<0.01
SOD(U/mL)	13.89±1.41^b^	18.67±0.43^a^	19.13±1.22^a^	17.82±0.50^a^	<0.05
CAT(U/mL)	41.67±4.14^C^	66.31±3.13^B^	64.52±2.56^B^	79.66±4.37^A^	<0.01

Peer data with different capital letters on shoulder labels indicated that the difference was extremely significant (*P* < 0.01), and without or with the same capital letters indicated that the difference was not significant (*P* > 0.01). Peer data with different lowercase letters on shoulder labels indicated that the difference was significant (*P* < 0.05), and no labeling or containing the same lowercase letters indicated that the difference was not significant (*P* > 0.05).

### Fertility rate of goose eggs

As can be seen from Fig. H, compared with group I, there is no significant difference in the fertilization rate of group II, III and IV, but group II, III, and IV show an upward trend in fertilization rate compared to group I. Notably, group II shows the most pronounced upward tendency among all groups.

**Fig. H:**
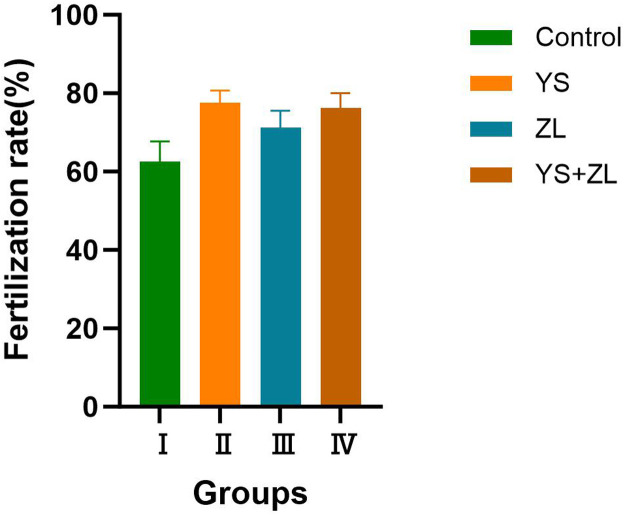
Effects of selenium yeast and zinc lysine supplementation on fertility rate of goose eggs.

## Discussion

Selenium yeast is a biological selenium formed by adding selenium during yeast culture, with the yeast fully absorbing and utilizing selenium when growing, organically combining selenium with protein and polysaccharide. Selenium plays an important role in preventing oxidative stress and has extremely high activity as a free radical scavenger and anti-cancer agent (Kieliszek, 2019). Selenium in animal semen mainly exists in the mitochondrial membrane, and selenium deficiency can lead to sperm cell membrane damage and decreased sperm motility (Brown and Arthur, 2001). Zinc lysine is a chelate formed by chemical bond between lysine and zinc ions. Its structure has good chemical stability and biological activity. However, zinc deficiency will lead to growth retardation, decreased feed utilization rate, decreased zinc concentration in tissues and organs, decreased activity of zinc-containing enzymes, and impaired protein synthesis in vivo (Vergnes et al., 1992; Samman et al., 1996). Sperm viability, the proportion of live sperm in a semen sample, is vital for reproduction. Only live sperm can fertilize an egg, and higher viability increases the chances of successful fertilization, directly impacting the fertilization rate. Thus, understanding and enhancing sperm viability are key to improving the reproductive performance of geese. The results of this study showed that adding YS and ZL to the diet could improve the sperm viability of goose. The reason may be that both YS and ZL have a promoting effect. Selenium, as one of the components of the outer membrane selenoprotein (SEP) of sperm mitochondria, reduces the lipid oxidation on the membrane and protects the cell membrane and mitochondria. ZL promotes testicular development, prevents spermatogenesis disorders, ensures the normal division and differentiation of spermatogonia, and jointly promotes the development of sperm cells. The above results show that adding YS and ZL to the diet can effectively promote sperm maturation and improve semen quality to some extent.

The reproductive system of male geese is mainly composed of testis and external genitalia. The former determines its spermatogenic ability, while the latter affects its mating behavior. The development process of male goose testis and external genitalia is influenced by varieties, nutrition, light, temperature, feeding methods and other factors. This experiment mainly explores the effects of YS and ZL on male goose testis tissue structure from the perspective of nutrition. The seminiferous tubule of testis is an important place for spermatogenesis (Cheng and Mruk, 2010). Leydig and Sertoli cells of testis are responsible for synthesizing and secreting reproductive hormones that regulate the development of reproductive system, and Sertoli cells also provide a stable environment for spermatogenesis (Johnson et al., 2008; van Bragt et al., 2008). Research points out pointed out that the effect of zinc deficiency on selenium metabolism is related to the change of sex hormone status in male animals, and zinc depletion leads to the decrease of selenium and glutathione peroxidase contents in testicular atrophy and testis (Behne et al., 1992). This study shows that the addition of YS and ZL can effectively improve the quality of seminiferous tubules and the number of spermatogonia, sertoli cells and germ cells in them. The reason may be that **SEP** in YS plays a role in regulating oxidative stress and apoptosis of germ cells in seminiferous tubules, while zinc in ZL plays a role in promoting spermatogenesis, preventing testicular oxidative stress and improving antioxidant status.

Selenium mainly functions in animals in the form of selenocysteine-containing SEP, zinc mostly participates in the metabolic process of organisms in the form of enzyme structure and enzyme activating factor (Cunnane, 1988), and hormones control testicular function through endocrine and adrenaline channels (Sofikitis et al., 2008). Testicular function determines spermatogenesis, so hormone signal is very important for successful spermatogenesis. Endocrine stimulation of spermatogenesis involves follicle stimulating hormone and luteinizing hormone, which acts through intermediate testosterone produced by leydig cells in testis (de Kretser et al., 1998). The main function of testis is to produce sperm. In this process, sertoli cells are very important for continuous spermatogenesis, providing physical and nutritional support for developing germ cells (Escott et al., 2014). Spermatogenesis begins when luteinizing hormone in pituitary activates leydig cells to produce testosterone, and testosterone then works with follicle stimulating hormone (O'Shaughnessy, 2014) to stimulate sertoli cells related to all aspects of spermatogenesis. Among the antioxidant abilities, SOD and GSH-Px activities can indirectly reflect the ability of scavenging free radicals, thus affecting the level of antioxidant function of male geese (Ray and Husain, 2002) and further affecting the growth and development of animals. The results of this study showed that adding YS and ZL to the diet can reduce the concentration of malondialdehyde (MDA) in the serum of male geese and effectively increase the concentrations of GSH-Px, T-AOC, CAT and SOD, which may be due to the fact that adding YS and ZL to the diet can reduce the level of free radicals in the body and increase the activity of antioxidant enzymes in some organisms. Enzyme activators in selenium and zinc in diet stimulate the metabolic process of reproductive hormone level, accelerate the occurrence process of reproductive hormone from hypothalamus-pituitary-gonad, and promote the increase of reproductive hormone level.

Reproductive performance reflects the egg-laying ability of livestock and poultry, and can be used as an important index to measure economic traits. The results of this study showed that adding YS and ZL to the diet could effectively exhibit a promoting trend for the fertilization rate of goose eggs. Although there were no significant differences between each treatment group, the fertilization rate after adding YS was 15 % higher than that of the control group. Studies have shown that l-selenomethionine can reduce the accumulation of ammonia and increase the rate of maturation and fertilization of porcine oocytes (Tareq et al., 2012). Research found that adding 0.2 mg/kg l-selenomethionine to the diet significantly improved the chick hatching rate, as well as selenium content in chicken egg yolk and embryo tissues (Wang et al., 2021). Because of its high bioavailability and actions upon antioxidant stress, YS has a more marked effect than ZL in the fertilization process, reducing oxidative stress and membrane damage during fertilization, and improving the fertilization rate. Zinc can promote the proliferation and differentiation of various cells in embryos during culture, regulate the metabolism in the body, and play an important role in promoting the growth and maturity of various organs and tissues of the body. Research showed that adding zinc to in vitro maturation medium improved the quality of oocytes and had a positive impact on the subsequent development of oocytes and embryos (Yao et al., 2023). The above results suggest that YS and ZL may be used as feed additives to improve the economic traits of laying geese.

## Conclusion

The results showed that adding 0.3 mg/kg selenium yeast and 106.05 mg/kg zinc lysine to the diet of geese can effectively improve semen quality, promote the development of the reproductive system, improve the spermatogenic ability of the testis, maintain a stable fertilization rate, and increase reproductive hormone levels and the serum antioxidant capacity.

## Declaration of competing interests

The authors declare that they have no known competing financial interests or personal relationships that could have appeared to influence the work reported in this paper.

## Appendix Supplementary materials

Image, application 1
