# Supplementary material for: Zinc lysine and selenium yeast can effectively improve the reproductive performance of Northeast white geese
Source: Poult Sci. 2025 Jan 31;104(3):104867. doi: 10.1016/j.psj.2025.104867 (PMC11847107; doi:10.1016/j.psj.2025.104867)
Supplement: Supplementary file 1 [file mmc1.pdf]

edanz 理文编辑
INNOVATIVE SCIENTIFIC SOLUTIONS

Certificate of Editing

Edited provisional title
Zinc lysine and selenium yeast can effectively improve the reproductive performance of Northeast white geese

Client name and institution
Jingchun Li, Heze Feng, College of Animal Science &veterinary medicine, Heilongjiang Bayi Agricultural University

Date Completed
2025-01-21

Identification code
611643

Certificate issued by
Edanz Editing China

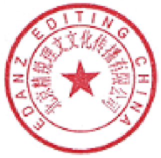

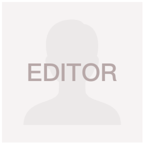

Expert Editor: Charles Allan
1992-PhD Biochemistry,Baker Medical Research Institute, Monash University
1987-BSc (Hons) Immunology and Pathology,Monash University
Paediatrics And Reproductive Medicine, Medical Physiology, Medical Biochemistry And Metabolomics

www.liwenbianji.cn

While this certificate confirms the authors have used Edanz's editing services, we cannot guarantee that additional changes have not been made after our edits.
